# Copy-number variation of the neuronal glucose transporter gene *SLC2A3* and age of onset in Huntington's disease

**DOI:** 10.1093/hmg/ddu022

**Published:** 2014-01-22

**Authors:** Angelica Vittori, Carlo Breda, Mariaelena Repici, Michael Orth, Raymund A.C. Roos, Tiago F. Outeiro, Flaviano Giorgini, Edward J. Hollox, the REGISTRY investigators of the European Huntington's Disease Network

**Affiliations:** 1 Department of Genetics, University of Leicester, Leicester, UK; 2 Cell and Molecular Neuroscience Unit, Instituto de Medicina Molecular, Lisbon, Portugal; 3 Department of Neurology, University of Ulm, Ulm, Germany; 4 Department of Neurology, Leiden University Medical Center, Leiden, The Netherlands; 5 Instituto de Fisiologia, Faculdade de Medicina da Universidade de Lisboa, Lisbon, Portugal; 6 Department of NeuroDegeneration and Restorative Research, University Medical Center Göttingen, Göttingen, Germany

## Abstract

Huntington's disease (HD) is a devastating neurodegenerative disorder which is inherited in an autosomal dominant manner. HD is caused by a trinucleotide CAG repeat expansion that encodes a polyglutamine stretch in the huntingtin (HTT) protein. Mutant HTT expression leads to a myriad of cellular dysfunctions culminating in neuronal loss and consequent motor, cognitive and psychiatric disturbances in HD patients. The length of the CAG repeat is inversely correlated with age of onset (AO) in HD patients, while environmental and genetic factors can further modulate this parameter. Here, we explored whether the recently described copy-number variation (CNV) of the gene *SLC2A3*—which encodes the neuronal glucose transporter GLUT3—could modulate AO in HD. Strikingly, we found that increased dosage of *SLC2A3* delayed AO in an HD cohort of 987 individuals, and that this correlated with increased levels of GLUT3 in HD patient cells. To our knowledge this is the first time that CNV of a candidate gene has been found to modulate HD pathogenesis. Furthermore, we found that increasing dosage of *Glut1*—the *Drosophila melanogaster* homologue of this glucose transporter—ameliorated HD-relevant phenotypes in fruit flies, including neurodegeneration and life expectancy. As alterations in glucose metabolism have been implicated in HD pathogenesis, this study may have important therapeutic relevance for HD.

## INTRODUCTION

Huntington's disease (HD, OMIM #143100) is a fatal autosomal dominant neurodegenerative disorder characterized by motor, psychiatric and cognitive dysfunction, with a mean age of onset (AO) of ∼40–50 years (1). The polymorphism underlying the disease is a CAG trinucleotide repeat in the *huntingtin (HTT)* gene, which leads to disease above a critical threshold of 36 repeats (2). The length of this repeat expansion accounts for up to 70% of the variability of AO, with which it is inversely correlated. Additional genetic factors are likely to explain a large proportion of the remaining variability of AO, and thus have the potential to modulate AO and other symptoms in HD (3). The identification of genetic modifier loci contributes to our understanding of HD pathogenesis and may ultimately facilitate the development of novel therapeutic interventions for this devastating disorder.

Aberrant glucose metabolism of the central nervous system (CNS) is a typical hallmark for several brain diseases. In the context of HD, 18F-fluorodeoxyglucose positron emission tomography scanning has revealed a loss of glucose uptake in the striatum and cortex of patients, remarkably before the onset of clinical symptoms of the disease (4). In asymptomatic HD gene carriers striatal metabolism is significantly decreased in the absence of atrophy, and the progression rate of HD shows a better correlation with the detected hypometabolism independent of CAG length (4–9). Interestingly, a 5-year follow-up study on asymptomatic HD gene carriers found that the caudate glucose metabolism was significantly decreased in patients that became symptomatic in the course of the study, and this change was not correlated with the CAG mutation length (10). In the same study, considering the relative caudate glucose metabolism along with CAG mutation length increases the regression coefficient for prediction of AO, indicating that glucose metabolism is a good predictor of disease onset (10,11). The role of the glucose metabolism in HD has also been demonstrated in several HD model studies. R6/2 HD model mice show an early and progressive metabolic impairment, which is significantly associated with lower glucose uptake, and is independent of cell loss (12). Furthermore, primary cortical neurones derived from HD140Q knock-in mice exhibit reduced levels of glucose uptake (13). Increasing glucose entry in glia was also found to reduce glia-induced pathology in fly models of HD (14). These studies suggest that dysfunction of glucose metabolism contributes to HD pathogenesis, and that its normalization may have therapeutic relevance in this disorder.

Glucose transport in humans is mediated either by active sodium–glucose co-transporters or by facilitative glucose transporters (GLUTs) (15). Several GLUT isoforms are expressed in the CNS, with GLUT1 (encoded by *SLC2A1*) and GLUT3 (*SLC2A3*) being the main transporters responsible for glucose uptake in the brain. GLUT1 is predominantly found at the blood–brain barrier while GLUT3 is primarily expressed in neurones, and shows higher glucose affinity and faster transport compared with GLUT1, characteristics that suit a high energy demand transporter (16).

While the homozygous knockout of *SLC2A3* causes embryonic lethality in mice, heterozygotes show abnormal spatial learning, social behaviour and working memory, as well as electroencephalographic seizures, although their motor abilities and coordination are normal (17). *SLC2A3* heterozygote mice show no differences in glucose uptake in the brain, suggesting that GLUT3 levels are not limiting in this particular model system (18). However, under hypoxic-ischaemia brain injury, *SLC2A3* heterozygote mice demonstrate spontaneous seizures and undergo enhanced brain apoptosis/necrosis, while control wild-type mice do not, suggesting that a difference in phenotype becomes more pronounced under stress (19).

In humans (and other primates), the *SLC2A3* gene is in a 129 kb region that has been tandemly duplicated to form *SLC2A14*, a testis-specific GLUT gene. Subsequent non-allelic homologous recombination events within the human population have resulted in an *SLC2A3* deletion allele and an *SLC2A3* duplication allele present at low frequencies in the European population (20). Therefore, copy-number variation (CNV) of *SLC2A3* has been observed, between one copy (heterozygous deletion) and three copies (heterozygous duplication).

In this study, we adopted a candidate gene approach, hypothesizing that CNV of a neuronal GLUT could influence HD AO. We reasoned that a decreased dosage of GLUT3 would make HD patients less capable of coping with situations where higher glucose demand is required, and thus lead to earlier disease onset. By extension, additional copies of *SLC2A3* may confer protection to patients, and lead to HD manifesting later in life. We found that increased dosage of *SLC2A3* was associated with an increase in GLUT3 in HD patients and a delay in AO in HD patients. Furthermore, we found that increased dosage of *Glut1*, the fruit fly orthologue of *SLC2A3*, is protective in HD model flies, underscoring the importance of GLUT3 in HD pathogenesis and potential future therapeutic interventions.

## RESULTS

### *SLC2A3* copy number modifies AO in HD

**Figure 1.**
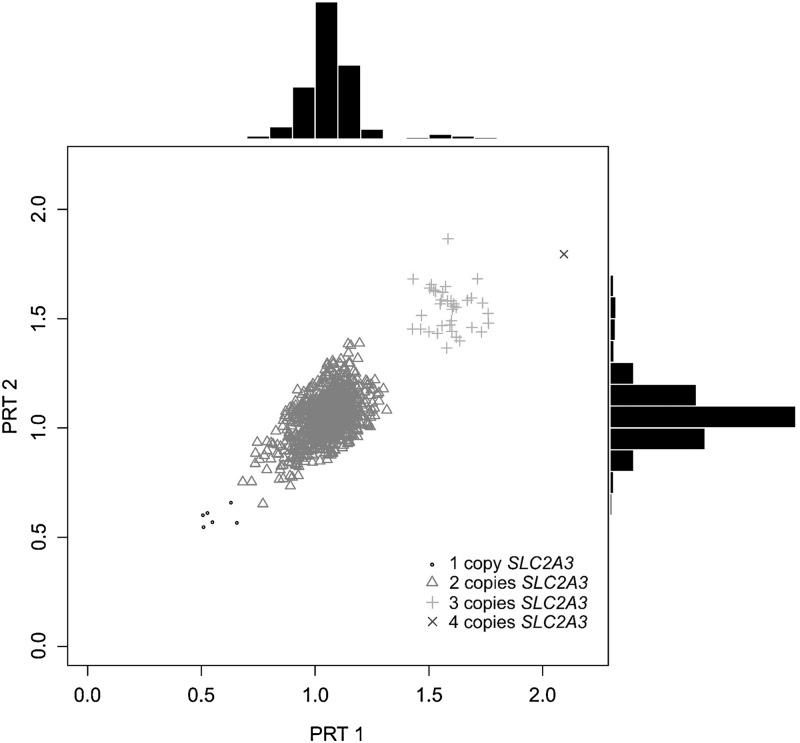
Scatterplot and histograms of raw PRT data. Raw duplicate PRT ratios for the 987 samples genotyped are shown. Points are distinguished to reflect the final *SLC2A3* copy number call, as shown in the legend. Summary histograms of the data are also shown.

**Figure 2.**
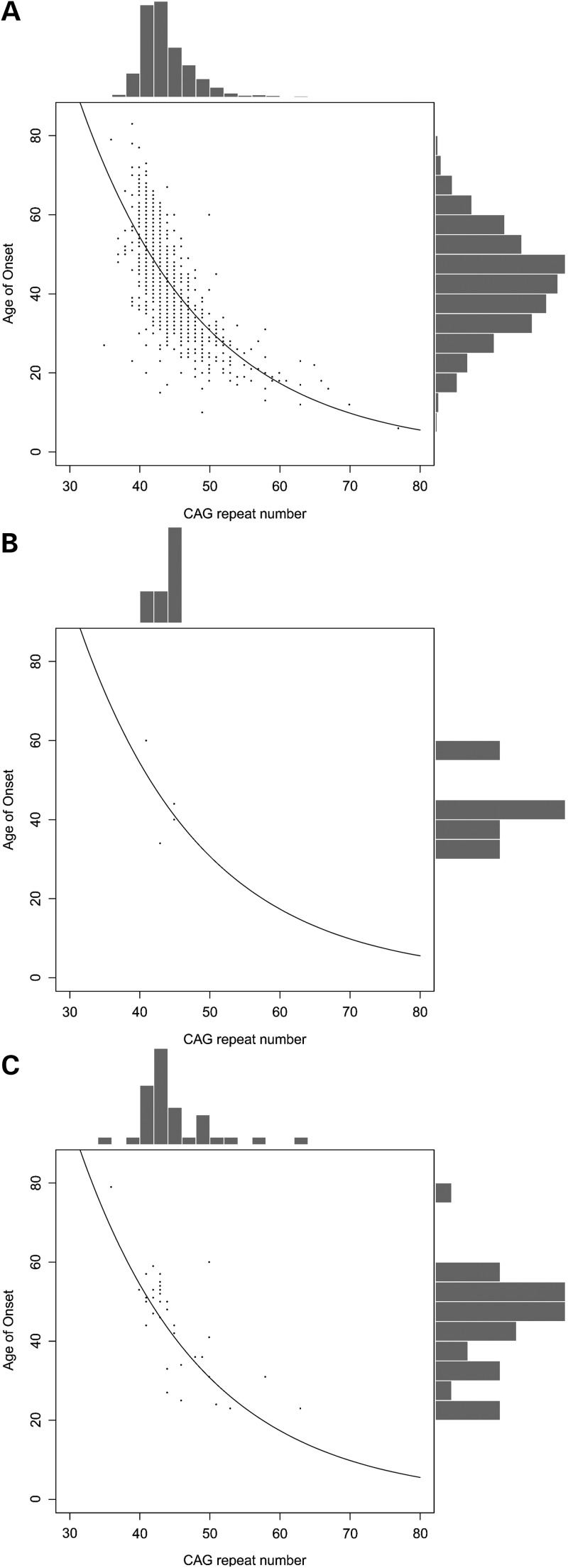
CAG length and AO of HD cohort, stratified by *SLC2A3* copy number. Scatterplots and histograms are shown for patients stratified by *SLC2A3* copy number. The exponential best fit line is shown, reflecting the linear best fit on log-transformed AO data. (**A**) Patients with *SLC2A3* copy number of two. (**B**) Patients with *SLC2A3* copy number of one. (**C**) Patients with *SLC2A3* copy number of three.

To investigate whether *SLC2A3* CNV might influence HD pathogenesis, we choose to genotype 987 HD patients for this genomic variation (Fig. 1). Diploid copy-number frequencies were determined (Table 1), and the duplication genotype frequencies (2, 3 and 4 copies) were found to be in Hardy–Weinberg equilibrium (*χ*^2^ = 1.16, *P* = 0.28). To assess the effect of copy number on AO, we initially plotted the distribution of CAG length and AO, stratified by *SLC2A3* copy number (Fig. 2). There is little obvious effect of the one copy genotype, with one copy individuals either side of the regression curve, but there is a noticeable difference in the distribution of three and four copy individuals, where 25/36 (69%) are above the regression curve, and therefore have a later AO than is predicted by CAG length alone.

**Table 1.** Diploid copy number frequencies in the HD cohort

*SLC2A3* copy number	Number
1 Copy	5
2 Copies	945
3 Copies	36
4 Copies	1
Total	987

We formally tested this by constructing a generalized linear model incorporating CAG length and *SLC2A3* copy number, which showed a significant positive association of *SLC2A3* copy number with log_2_AO (*P* = 0.022). This association is not dependent on the threshold calling of copy number between one and two copies (Table 2). Indeed altering the threshold, so that fewer samples are called at one copy improves the significance level slightly to 0.015. The modest significance value reflects the small overall effect on AO in the HD population, and this is due to the low frequency of the deletion (0.25%) and duplication alleles (1.9%). Indeed, the effect on individual AO is pronounced, using the regression model the increase in AO per copy can be estimated at ∼3 years [95% confidence interval (CI) 0.4–5.67, Table 3].

**Table 2.** Threshold for calling *SLC2A3* 1 copy patients and significance of association

PRT threshold for calling 1 copy	0.6	0.65	0.7	0.75
Number of 1 copy patients called	3	5	5	7
*P-*value	0.015	0.022	0.022	0.019

**Table 3.** Effect of *SLC2A3* copy number on AO

	Mean (95% CI) (years)	*P*-value
Effect per extra CAG repeat	−2.34 (−2.24 to −2.45)	<5 × 10^−6^
Effect per extra copy of *SLC2A3*	2.98 (0.40–5.67)	0.022

### CNV of *SLC2A3* in HD patient lymphoblastoid cells alters protein levels of GLUT3

We then asked whether *SLC2A3* copy number affected GLUT3 protein levels by a gene dosage effect. Although predominantly expressed in neurones, *SLC2A3* is expressed in other tissues and also in leukocytes, including B cells (21). Unfortunately, neuronal tissue or cell lines from patients with different *SLC2A3* copy numbers were not available. However, lymphoblastoid cell lines (LCLs), derived from Epstein–Barr virus-immortalization of B cells from peripheral blood, were available, and therefore chosen as a model to investigate the gene dosage effect of *SLC2A3*.

**Figure 3.**
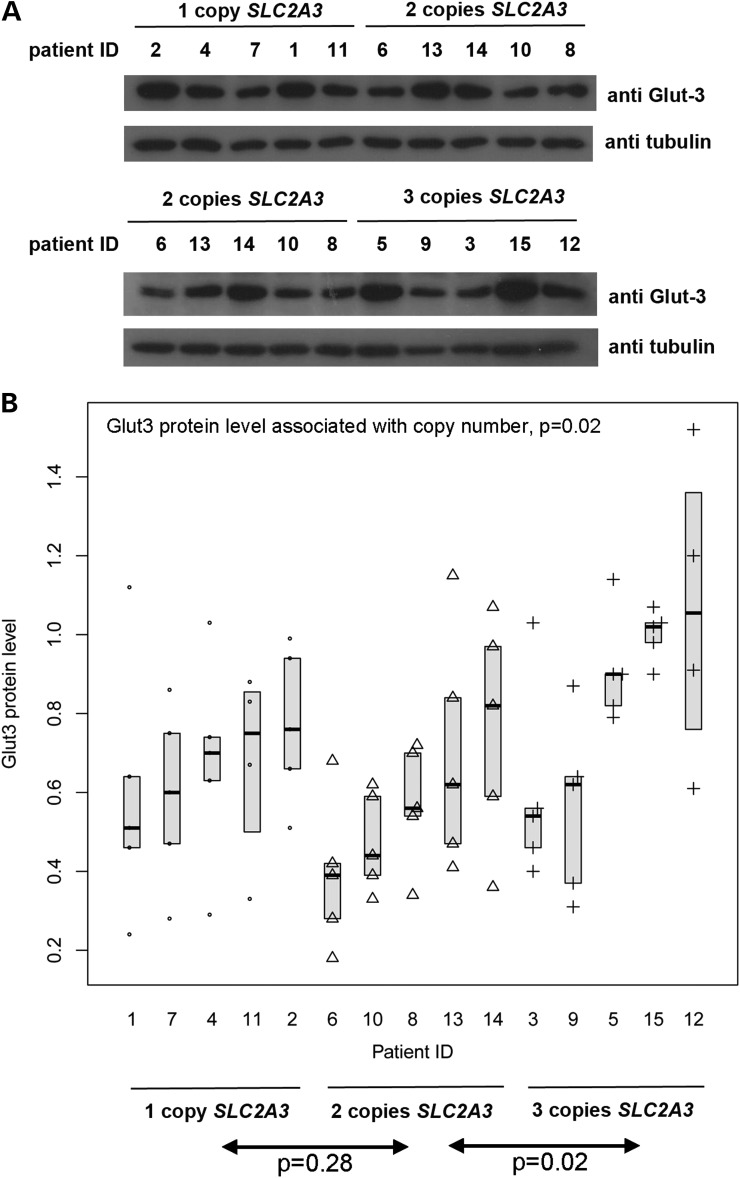
Relationship between *SLC2A3* copy number and GLUT3 protein levels in LCLs from HD patients. (**A**) Two representative western blots of protein extracted from 20 LCLs from HD patients. In each example, the upper panel shows detection of GLUT3 protein revealed by anti-GLUT3 antibody, and the lower panel shows detection of tubulin as a loading control. (**B**) Graph summarizing data from quantification of repeated western blot experiments. Individual points represent individual measurements, with point shape reflecting *SLC2A3* copy number of the cell line (circle = 1 copy, triangle = 2 copies, cross = 3 copies). Boxes show median and interquartile range. GLUT3 protein level is shown as a value relative to the level of tubulin detected in the same gel lane.

For each of *SLC2A3* copy numbers 1, 2 and 3, five LB cell lines were grown and total protein extracted. Semi-quantitative immunoblotting was used to estimate the amount of GLUT3 protein in the cells, relative to levels of tubulin as a control. For each cell line, measurements from at least four replicate blots were analysed (two representative blots are shown in Fig. 3). Considerable variation exists within each copy number category, both at the biological level between cell lines from different patients with the same copy number, and at the technical level due to the semi-quantitative nature of the assay. To fully allow for this variation, we employed a mixed effects linear regression model to explore the association between GLUT3 protein level and *SLC2A3* copy number. Pairwise comparison showed a significantly higher expression level in three copy individuals compared with two copy individuals (*P* < 0.001) but not in two copy individuals compared with one copy individuals (*P* = 0.28). This is consistent with our observation from the HD patient cohort that three *SLC2A3* copies contributes most to the neuroprotective effect, but this could also be due to a lack of power in either experiment. Indeed, a significant linear correlation between copy number and GLUT3 expression level was seen (*β* = 0.09, *P* = 0.02).

### Altered dosage of the GLUT3 orthologue in *Drosophila* modulates HD phenotypes

**Figure 4.**
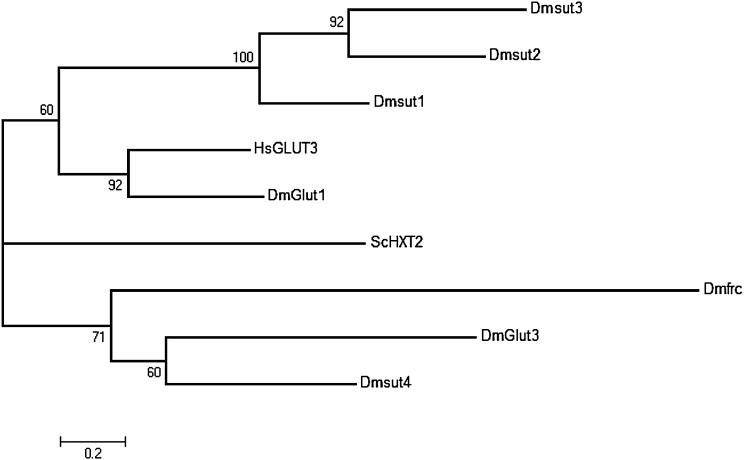
Phylogenetic tree of human GLUT3 protein (HsGLUT3) and candidate homologues in *Drosophila*. Numbers at nodes represent percentage of 500 bootstrap trees supporting that node. Scale bar represents 0.2 amino acid substitutions per site. ScHXT2 is a hexose transporter from *S. cerevisiae* and is considered the out-group.

To recapitulate the effect of *SLC2A3* CNV on HD, we next turned to an established and well-characterized *Drosophila* model of HD (22) which we have extensively employed in other studies (23–28). This model uses the bitransgenic UAS/GAL4 system to drive pan-neuronal expression of an exon 1 mutant HTT fragment (HTT93Q) using the *elavGAL4* driver, and yields several disease-relevant phenotypes, including degeneration of rhabdomeres (photoreceptor neurones), reduced lifespan, and impaired eclosion of adult flies from the pupal case. To identify the orthologue of the human GLUT3 protein, we searched two databases for predicted GLUTs in *Drosophila*. Seven candidate protein sequences were identified, which were aligned and a phylogenetic tree constructed using a maximum-likelihood approach (Fig. 4). The *Drosophila* protein GLUT1 is the clear orthologue of human GLUT3, with an amino acid identity of 46%, and is its functional homologue since it is the neurone-specific facilitative GLUT in *Drosophila*.

**Figure 5.**
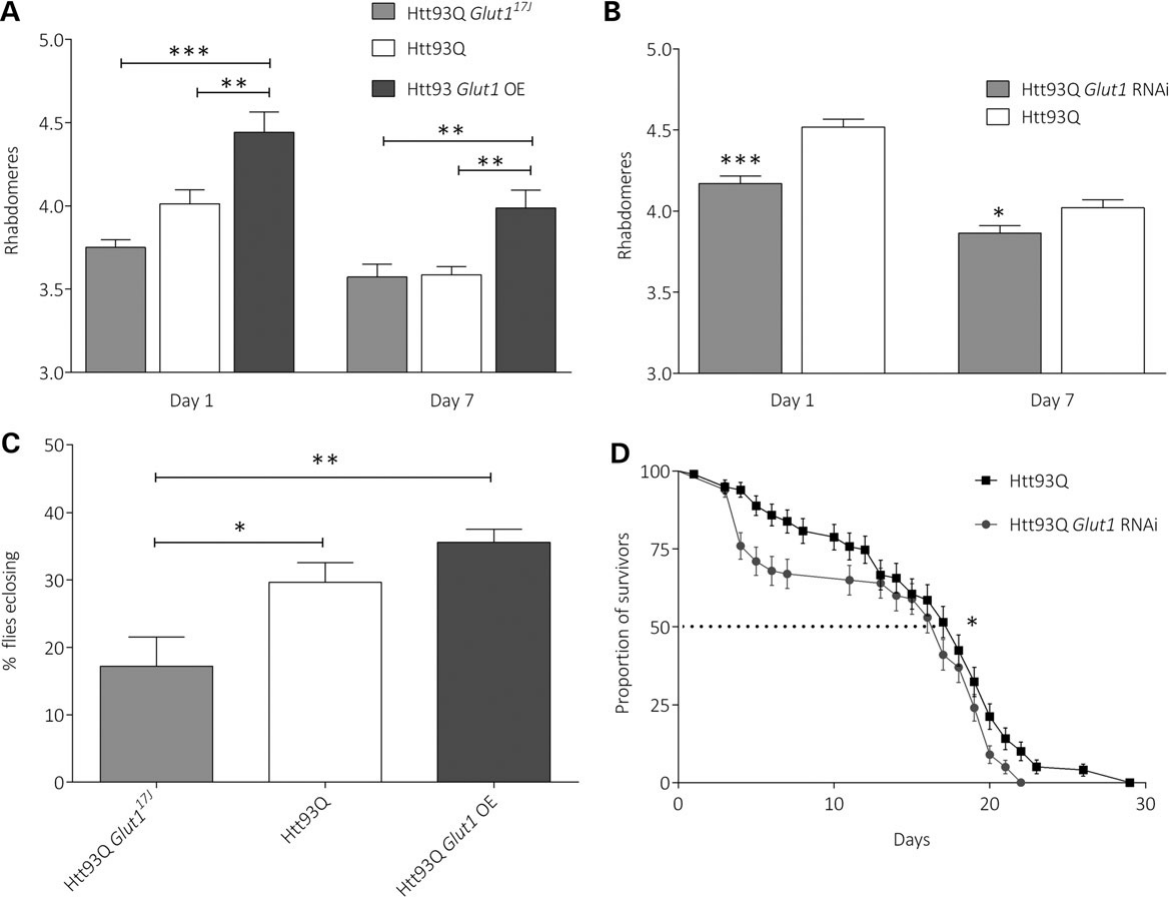
Increased pan-neuronal dosage of *Glut1* in *Drosophila* improves HD phenotypes. (**A**) Htt93Q *Glut1*^17J^ flies enhanced rhabdomere loss in Day 1 Htt93Q flies, while *Glut1* overexpression protected against neurones loss at Days 1 and 7. *n* = 8–15. Statistical comparisons by ANOVA and *post hoc* test (**P-value < 0.01; ***P-value < 0.001). Mean ± SEM. (**B**) Quantification of rhabdomeres per ommatidium in Htt93Q and Htt93Q *Glut1* RNAi flies at Days 1 and 7 after eclosion. *N* = 9–15 flies per genotype. Statistical comparisons by ANOVA and *post hoc* tests versus Htt93Q flies (*P-value < 0.05; ***P-value < 0.001). Mean ± SEM. (**C**) Htt93Q *Glut1*^17J^ flies showed a decreased adult emergence from the pupal case compared with Htt93Q and Htt93Q *Glut1* OE flies. *n* ≥ 1000 progeny for each cross. Statistical comparisons by ANOVA and *post hoc* test (*P-value < 0.05; **P-value < 0.01). Mean ± SEM (**D**) *Glut1* down-regulation decreased median survival of adult HD flies. The number of flies surviving from each cohort was determined every day. *n* = 100 animals per genotype. Statistical analysis by Kaplan–Meier survival curve analysis with log-rank test (*P-value < 0.05).

We thus interrogated GLUT1 function in this fly model of HD via several approaches. First, we confirmed that *Glut1* expression levels were modulated in these transgenic lines using quantitative real-time polymerase chain reaction (QPCR) analysis (Supplementary Material, Fig. S1). We found that RNAi knockdown reduced *Glut1* mRNA expression by ∼35% (*P* < 0.05), while overexpressing *Glut1* using the *Glut1^d05758^* overexpression line significantly increased expression levels by ∼78% (*P* < 1 × 10^−4^). Having validated the specificity of the transgenic lines, we next investigated the effects of GLUT1 manipulation on disease-relevant phenotypes in Htt93Q flies. First, we employed a robust readout of neurodegeneration—the degeneration of the photoreceptor neurones (rhabdomeres) (Fig. 5A and B). We find that either a loss-of-function mutation in the *Glut1* gene (*Glut1^17J^*), or RNAi knockdown of this gene, enhances rhabdomere loss in Htt93Q flies, though only in the case of RNAi is the effect significant. We also find that *Glut1* overexpression—via the *Glut1^d05758^* allele (29)—significantly rescues this neurodegeneration (Fig. 5A). Strikingly, these results parallel the HD AO observations above—increased dosage of *Glut1* expression is correlated with amelioration of disease phenotypes.

We next asked whether *Glut1* dosage in HD flies could modulate general viability and health phenotypes in these animals by scoring eclosion frequency and lifespan. Htt93Q *Glut1^17J^* flies exhibit reduced eclosion frequency compared with Htt93Q (*P* < 0.05) or Htt93Q *Glut1*-overexpressing flies (*P* < 0.01) (Fig. 5C), and that Htt93Q *Glut1*-overexpressing flies have increased eclosion versus Htt93Q flies, though this is not significant. We also observed that Htt93Q *Glut1* RNAi flies have a significantly shorter lifespan compared with Htt93Q flies (*P* < 0.05) (Fig. 5D), while *Glut1* overexpression has no effect on this metric (data not shown). In total, these data support our neurodegeneration data, and suggest that increased dosage of *Glut1* ameliorates disease phenotypes in HD flies.

## DISCUSSION

Taken together, our data from human genetic analysis, molecular biology and a *Drosophila* HD model strongly support the hypothesis that increased dosage of *SLC2A3* ameliorates HD phenotypes, which may have important therapeutic ramifications. To our knowledge this is the first example indicating that a CNV event can be a genetic modifier of AO in HD. While it is known that gene deletion and duplication can affect neurodegeneration, in the past this has been limited to a small subset of a complex neurodegenerative disorders that show Mendelian inheritance, for example the triplication of α-synuclein or large deletions of the Parkin gene causing Parkinson's disease (30,31). The role of CNV with alleles at polymorphic frequencies in modifying the susceptibility to neurodegenerative disease remains unclear.

Many other studies have identified possible genetic variants that appear to affect the AO in HD patients; the most frequently identified variants are single nucleotide polymorphisms (SNPs) often of uncertain functional effect (32). Indeed, many early studies showing putative effects of SNPs on HD AO have not been replicated by larger studies, suggesting that most of the initial reports, often on small patient cohorts, are false positives (33,34). In this study, we analysed almost 1000 patients from the REGISTRY investigators of the European Huntington's Disease Network (EHDN) for a rare CNV in the *SLC2A3* gene. The association has a modest significance (*P* = 0.022) reflecting the low frequency of the deletion and duplication alleles (0.25 and 1.9%, respectively), but, importantly, carrying a duplication allele (3.6% of patients in our study) delays the AO of clinical symptoms of HD by 3 years. The low frequency of both duplication and deletion alleles of *SLC2A3* poses a particular challenge in genetic association studies. In this study, despite a strong size of effect attributed to *SLC2A3* CNV, the statistical support for such an effect is modest. This has two consequences. First, it illustrates the importance, if feasible, of recapitulating the CNV in a model system, to provide further biological support for a posited effect. Second, it emphasizes the importance of large patient cohorts in identifying and confirming genetic modifier effects.

A recent study identified the *SLC2A3* CNV, with allele frequencies of the deletion and duplication consistent with our observed frequencies: 1% deletion frequency and 2.2% duplication frequency in a Swedish population (20). A replicated case–control study showed deletion of *SLC2A3* was strongly protective against rheumatoid arthritis (odds ratio 0.442), and it was suggested that the role of GLUT3 as a GLUT in B cells and chondrocytes may provide a biological basis for this observation. The authors also showed that the CNV alleles were not effectively tagged by flanking SNPs with a minor allele frequency >5%. These data suggest that disease effects of CNV at this locus are invisible to SNP-based genome-wide association studies, at least at the usual current study sizes. Our studies suggest that HD progression could be affected by GLUT3 expression level, and functionality as in neurones glucose uptake is mainly dependent on this transporter. Indeed, a post-mortem brain study found a significant reduction in GLUT3 levels in the caudate of Grades 1 and 3 HD brain, but not in the cortex (35).

Our observations highlight the importance of neuronal glucose metabolism in neurodegenerative disease and suggest novel candidate therapeutic avenues. It will be now critical to study the potential for pharmacological modulation of GLUT3 function or expression, as well as uncovering more general approaches for increasing glucose import into neuronal populations sensitive to degeneration or dysfunction in susceptible regions of the HD brain. While our study is limited to HD, we believe that these findings will have direct relevance to the pathogenesis of other neurodegenerative disorders, and may ultimately inform future therapeutic strategies for a broad variety of these related diseases.

## MATERIALS AND METHODS

### HD patient DNA samples and *SLC2A3* genotyping

The study cohort comprised of 987 unrelated individuals of European ancestry with manifest HD enrolled in the EHDN ‘REGISTRY’ study (36). ‘REGISTRY’ is a multi-centre, multi-national observational study, which catalogues data in a wide range of the European HD population (http://www.euro-hd.net/html/registry last accessed 25 January 2014). Experienced investigators estimate the age at onset based on the integration of data from the patient's history, collateral history of family or carers and the examination of the patient. Manifest HD was defined as carrying the *HTT* CAG repeat expansion mutation (≥36) and having a motor score with a diagnostic confidence of four on the UHDRS motor scale (37). Clinical data on AO and mutant CAG repeat size were provided. In our cohort, AO was defined as the AO of any symptom of HD. In most patients (620), these were motor symptoms, with psychiatric symptoms accounting for the second largest grouping (169).

*SLC2A3* copy number was typed on these individuals using a paralogue ratio test (PRT), which is a form of quantitative PCR that uses the same primer pair for test and reference amplicons (38). The PRT assay, termed ‘P1’, was developed and extensively validated previously (20). We used a fluorescently labelled primer to allow electrophoresis and subsequent fluorescent detection of products using an Applied Biosystems ABI 3130XL capillary electrophoresis machine. Each sample was typed twice, an estimate of the ratio of test:reference peak areas made and repeated if the coefficient of variation of the two values was >0.15. The raw results of the duplicate measurements are shown in Figure 1.

We called integer copy number, blind to clinical status or *HTT* CAG repeat number, using the PRT ratio thresholds derived by manual inspection of the data clustering. The single outlier with a high PRT ratio could either be a homozygous duplication (*SLC2A3* copy number 4) or be a heterozygous deletion of the reference region, but following confirmation of presence of the duplication allele by a breakpoint-specific PCR (39) this was called as a *SLC2A3* four copy individual.

### HD lymphoblastoid cell culture and immunoblotting

Lymphoblastoid cell lines (LCLs) were established from peripheral blood of HD patients by BioRep (Milan, Italy) for the EHDN. Five LCLs were selected from each group of individuals (carrying 1, 2 and 3 copy number of *SLC2A3*), which were previously genotyped in our study. The cell lines were maintained at 0.5–1 × 10^6^ cell/ml concentration in RPMI 1640 with GlutaMAX™ (Life Technologies) with 10% fetal bovine serum, 100 U/ml penicillin and 100 μg/ml streptomycin, at 37°C in a 95% air/5% CO_2_ atmosphere. Cell pellets containing 20 × 10^6^cells were washed two times with sterile phosphate buffered saline and then lysed in 200 µl of lysis buffer (40). Lysates were centrifuged at 13 000 revolutions per minute for 10 min at 4°C. Supernatants were collected, and protein concentration was determined by the Bradford method. Samples were stored at −80°C until analysed. Ten micrograms of total cell lysate were separated on a 10% sodium dodecyl sulphate polyacrylamide gel and transferred to a polyvinylidene difluoride membrane. Incubation with primary antibodies was overnight at 4°C, using polyclonal rabbit anti-GLUT3 antibody (1:4000; ab15311, Abcam) or mouse anti-tubulin (1:1000; sc-8035, Santa Cruz Biotechnology). Blots were developed using horseradish peroxidase-conjugated secondary antibodies (1:10 000; PI-1000 anti-rabbit, Vector Laboratories, and 1:10 000; PI-2000 anti-mouse, Vector Laboratories) and the ECL system (SuperSignal West Dura Extended Duration Substrate, Thermo Scientific). Semi-quantitative analysis of immunoblot data was assessed using ImageJ software (National Institutes of Health).

### Identification of GLUT3 *Drosophila* homologue

Candidate *Drosophila* homologues of human GLUT3 were identified by a search of UniProt and by blastp analysis of *Drosophila* protein sequences in the non-redundant database. The protein sequence HXR2 GLUT of *Saccharomyces cerevisiae* was used as an out-group. Clustal Omega was used to align the amino acid sequences, and MEGA5.2 (41) was used to generate a maximum-likelihood tree, with 500 bootstrap replicates, the Jones–Taylor–Thornton model of protein evolution (42), with uniform rates across sites.

### *Drosophila* stocks

Flies were raised on standard maize media, in a 12 h light, 12 h dark cycle at 25°C. The *Glut1^d05758^* and *Glut1^17J^* alleles and *elav-GAL4* (c155) driver stocks were obtained from the Bloomington Stock Center (IN, USA). The *Glut1* RNAi transgenic line (108683) was obtained from the Vienna *Drosophila* RNAi Centre (Austria). The *w;+; UASHttQ93* exon1 line (43) was a gift from J.L. Marsh and L. Thompson (University of California, Irvine).

### Pseudopupil analysis

The number of visible rhabdomeres per ommatidium was scored from 40 to 100 ommatidia per fruit fly, with 8–15 flies examined per genotype at Days 1 and 7 post-eclosion. Heads were removed from the body and fixed to a microscope slide using fingernail polish. Rhabdomeres were visualized at 500× magnification using either a Nikon Optiphot-2 or an Olympus BH2 microscope.

### Eclosion and longevity analyses

The eclosion rate was scored from 10 independent crosses between five males carrying the *elav-GAL4* driver and five virgin females carrying the transgenes of interest. The numbers of males and females were counted every day until all of the viable F1 progeny had enclosed. The adult emergence percentage was calculated as a ratio between female and all progeny, where the females expressed the transgene of interest (Htt93Q). Lifespan was assessed on ∼100 female flies for each strain analysed. Flies were collected within 24 h of emergence, divided into groups of 10 per vial and kept at 25°C. According to the genotype of interest, the flies were transferred daily or every 3-4 days in a new tube and the dead flies were scored.

### Statistics

Association of *SLC2A3* copy number with HD AO was tested by generating a generalized linear model using SPSS 20 (IBM), with CAG as a continuous predictor, *SLC2A3* copy number as an ordinal predictor and log_2_AO as the continuous response variable, using Wald Type III analysis of variance (ANOVA) statistics. Association of *SLC2A3* copy number with protein levels was tested by a mixed effects linear model, after confirmation that the distribution of GLUT3 levels did not depart from a Gaussian distribution (*P* = 0.17, Shapiro–Wilks test). Statistical analyses of the *Drosophila* data were carried out using ANOVA where applicable with Newman–Keuls *post hoc* tests using Statistica (StatSoft Ltd.) or Prism 6 (GraphPad Software). For longevity analysis, survival curves were generated, and data were analysed by using the Kaplan–Meier method and statistical significance ascertained using log-rank statistics software (GraphPad Software).

## SUPPLEMENTARY MATERIAL

Supplementary Material is available at *HMG* online.

## FUNDING

A.V. was supported by a FCT PhD studentship (SFRH/BD/4764/2008) awarded to T.F.O., F.G. and E.J.H. Both E.J.H. and F.G. were supported by MRC New Investigator awards (GO801123 to E.J.H. and G0700090 to F.G.). T.F.O. is supported by the DFG Center of Nanoscale Microscopy and Molecular Physiology of the Brain (CNMPB). Funding to pay the Open Access publication charges for this article was provided by the Research Councils UK Open Access fund at the University of Leicester.

## Supplementary Material

Supplementary Data
