# Supplementary material for: Copy-number variation of the neuronal glucose transporter gene SLC2A3 and age of onset in Huntington's disease
Source: Hum Mol Genet. 2014 Jan 22;23(12):3129–37. doi: 10.1093/hmg/ddu022 (PMC4030768; doi:10.1093/hmg/ddu022)
Supplement: Supplementary Data [file supp_23_12_3129__index.html]

Copy-number variation of the neuronal glucose transporter gene SLC2A3 and age of onset in Huntington's disease — Copy-number variation of the neuronal glucose transporter gene SLC2A3 and age of onset in Huntington's disease — Supplementary Data 

# Copy-number variation of the neuronal glucose transporter gene *SLC2A3* and age of onset in Huntington's disease

## Supplementary Data

Supplementary Data

**Files in this Data Supplement:**

- Supplementary Figure 1 - pdf file
- Supplementary Data - txt file
