# Supplementary material for: Copy-number variation of the neuronal glucose transporter gene SLC2A3 and age of onset in Huntington's disease
Source: Hum Mol Genet. 2014 Jan 22;23(12):3129–37. doi: 10.1093/hmg/ddu022 (PMC4030768; doi:10.1093/hmg/ddu022)
Supplement: Supplementary Data [file supp_ddu022_ddu022supp_fig1.pdf]

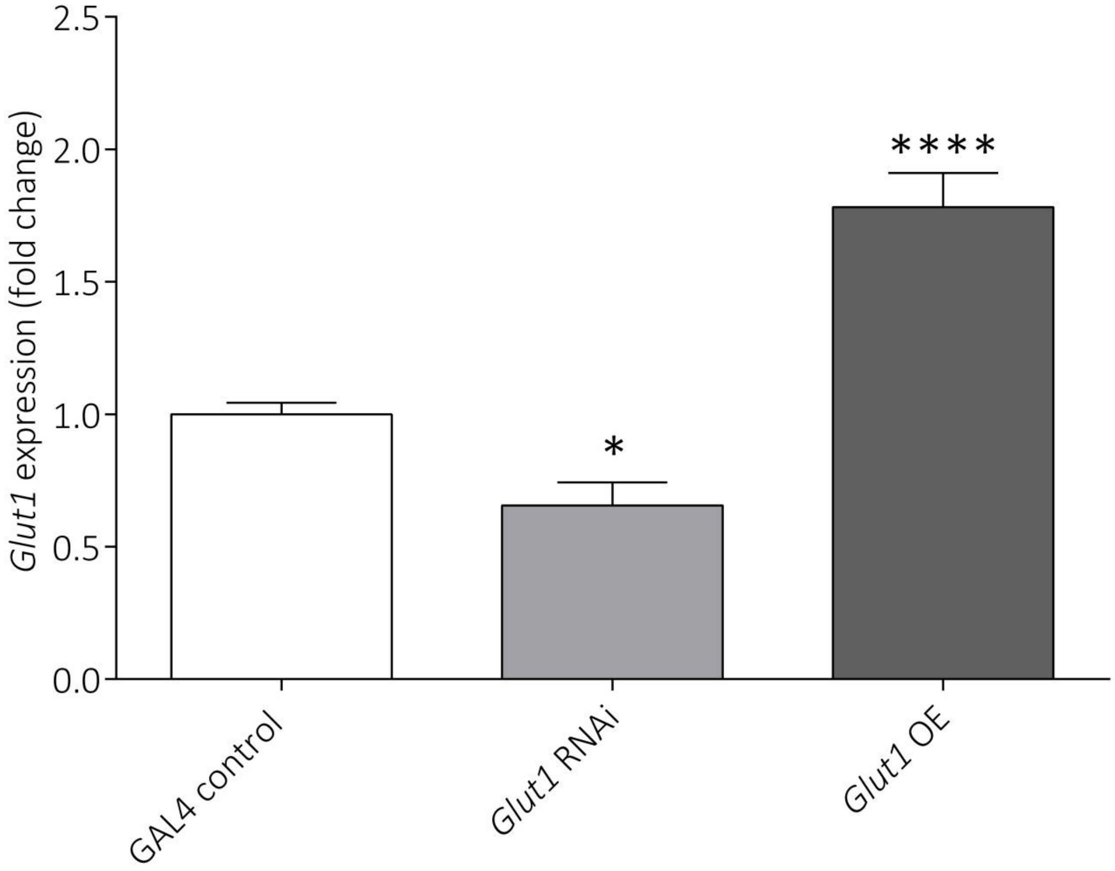

Figure S1. Relative quantification of *Glut1* expression levels in the transgenic fly strains.

Relative quantification of normalised ΔCt mean (± SEM) for the GAL4 control, *Glut1* RNAi and *Glut1* overexpressing (OE) flies. n ≥ 30 flies per each strain. Statistical comparisons by ANOVA and post hoc tests versus GAL4 control flies. (*p value <0.05; **** p value < 0.0001).
